# Integrating experimental paper analysis into traditional ancient book restoration: A case study of the Chinese medical text Yi Men Pu Du

**DOI:** 10.1371/journal.pone.0360242

**Published:** 2026-10-09

**Authors:** Chen Li, Sijia Gao, Meng Li

**Affiliations:** Institute of Chinese Medicine Information, China Academy of Chinese Medical Sciences, Beijing, China; UKM: Universiti Kebangsaan Malaysia, MALAYSIA

## Abstract

**Background:**

Ancient Chinese books represent irreplaceable cultural heritage, yet many suffer severe deterioration from acidification, insect damage, and mechanical degradation. Traditional restoration practices rely heavily on empirical experience, with limited integration of scientific testing. This study demonstrates how systematic experimental paper analysis can be embedded into the traditional restoration workflow of a critically damaged ancient Chinese medical book.

**Methods:**

The ancient medical text *Yi Men Pu Du* (1739), classified as Grade 1 (critical) damage under Chinese heritage conservation standards, underwent comprehensive paper characterization before, during, and after restoration. Fiber composition was identified by optical microscopy (GB/T 4688–2020). Physical properties—grammage (ISO 536:2012), thickness (GB/T 451.3-2008), and tensile strength (GB/T 22898–2008)—were measured for both original and repair papers. Surface and cold-extraction pH were determined (GB/T 13528–2015; GB/T 1545–2008). A Ca(OH)_2_ deacidification protocol (1 g:200 ml) was applied in three successive stages.

**Results:**

Fiber analysis revealed 100% bark fiber in the cover and 100% bamboo fiber in the text leaves, directly guiding repair paper selection. Pre-restoration surface pH ranged from 5.1 to 5.4, confirming severe acidification. Progressive deacidification raised pH to 7.4–7.7. Thickness monitoring identified excessive post-restoration thickness (195 μm) caused by overly concentrated pulp, which was resolved by reducing the pulp ratio from 5 g/400 ml to 0.5 g/400 ml. Bamboo repair paper exhibited 29.95%–36.66% higher longitudinal tensile strength than bark papers.

**Conclusion:**

Systematic experimental analysis transformed an experience-based restoration into an evidence-based practice, providing quantitative guidance for material selection, deacidification, and process optimization. This methodological framework is transferable to other ancient book conservation projects.

## 1. Introduction

Ancient books are indispensable carriers of human civilization. In China, the tradition of handmade papermaking spans over two millennia, producing papers of remarkable durability—exemplified by the *Dunhuang Manuscripts*, which have survived for more than 1,600 years [1]. Chinese handmade paper is crafted from plant-based raw materials and classified by fiber composition into bark (vellum) paper, hemp paper, and bamboo paper [2]. As papermaking technology evolved, production costs decreased and raw materials shifted from hemp and bark fibers to predominantly bamboo fiber [3]. During the Republican period (1912–1949), however, the widespread adoption of Western acid-based processing yielded papers with significantly reduced longevity [4]. Over the past century, machine-made papers from this era have deteriorated far more rapidly than handcrafted papers from the Ming (1368–1644) and Qing (1644–1912) dynasties, posing formidable challenges for conservation specialists.

Paper deterioration is driven primarily by acid hydrolysis of cellulose, a process accelerated by acidic additives introduced during manufacture and by atmospheric pollutants absorbed during long-term storage [5,6]. Acid-catalyzed depolymerization of cellulose chains progressively weakens paper until it becomes brittle and friable [7]. Deacidification treatments—particularly alkaline agents such as calcium hydroxide (Ca(OH)_2_)—have been widely adopted to neutralize existing acidity and deposit an alkaline reserve for long-term protection [8,9]. Beyond chemical stabilization, appropriate material selection is equally critical: repair papers must be compatible with the original substrate in fiber composition, grammage, and mechanical properties to ensure structural and aesthetic integrity [10].

Despite advances in conservation science, the integration of systematic experimental analysis into practical restoration workflows remains limited in many countries. In China, recent national initiatives have elevated ancient book restoration as a component of intangible cultural heritage conservation [11]. However, traditional restoration practice continues to rely predominantly on empirical judgment, with scientific testing applied only sporadically. This gap between laboratory research and workshop practice represents a significant obstacle to evidence-based conservation.

The present study addresses this gap through the restoration of *Yi Men Pu Du*, a valuable ancient Chinese medical book dating to 1739. The book was classified as Grade 1 (critical) damage—the most severe category under Chinese heritage conservation standards—and required comprehensive intervention including fiber analysis, deacidification, pulp repair, and structural reinforcement. The restoration was guided by four core principles established in the national standard *Standard for the Restoration and Control of Ancient Books* (GB/T 21712−2008) [12]: (i) “preserving the old as it was” (修旧如旧), safeguarding historical integrity; (ii) “minimal intervention,” restricting modifications to those necessary for stabilization; (iii) “reversibility,” ensuring all materials can be safely removed in the future; and (iv) “maintaining the status quo,” preserving the artifact without speculative additions.

The objective of this study was to demonstrate how systematic experimental characterization—encompassing fiber identification, physical property testing, and chemical analysis—can be integrated into each stage of a traditional restoration workflow, transforming experience-based craft into evidence-based practice. The methodological framework developed here is intended to be transferable to other ancient book conservation projects.

## 2. Materials and methods

### 2.1. Damage assessment

The damage grade of *Yi Men Pu Du* was evaluated in accordance with WH/T 22–2006, *Standard for Distinction of Disrepair of Ancient Books and Special Collections* [13]. In this standard, Grade 1 damage (一级破损) represents the most severe category, corresponding to critical damage requiring salvage restoration (Table 1). This grading convention—where a lower grade number indicates greater severity—is specific to Chinese cultural heritage conservation standards.

**Table 1:** Damage grade classification of ancient books (based on WH/T 22-2006 [13]).

Grade	Typical characteristics	Restoration priority
Grade 1 (Critical)	Paper embrittlement and fragmentation, mildew agglomeration, carbonization, severe acidification, insect damage >50%, extensive text loss	Salvage restoration
Grade 2 (Severe)	Large-area page loss, spine fracture, multi-page adhesion, insect-bore holes penetrating multiple pages	Emergency restoration
Grade 3 (Moderate)	Corner loss, partial tears (length < 1/2 page), water stains causing partial text blur, single-page insect-bore holes	Priority restoration
Grade 4 (Mild)	Edge wear, minor stains, single-page small creases, cover fading	Routine maintenance

*Yi Men Pu Du* was classified as Grade 1 (critical) damage. The standard criteria quoted include: “4.2 The leaf paper is seriously aging, resulting in reduced mechanical strength, slagging, cracks and breakage when turned over,” and “4.4 More than 50% of leaf area is moth-eaten, with more than 80% moth-eaten leaves throughout” [13]. The book exhibited severe deterioration: all leaves were dirty and severely acidified, with wormholes present throughout the interior pages. Insect damage had caused hardening of the spine, with insect excrement and eggs leading to page adhesion. Serious brittle cracking was evident, with loss of frame support causing acidification at the upper and lower margins of page layouts, accompanied by tears and fractures. Additionally, traces of burning were present on the front cover and footing, with slight dehydration and carbonization on the center of the front page, and numerous fragments had detached from the upper corner of the book block.

### 2.2. Restoration protocol

Following extensive deliberation among restoration team members and external experts, a comprehensive restoration plan was developed, as illustrated in Fig 1. The plan combined two primary techniques: the FeiTuo method (飞托法, a support-mounting technique for fragile leaves) and the manual drip-pulp method (手工滴浆法, a technique involving the application of diluted pulp suspension onto damaged areas).

**Fig 1:**
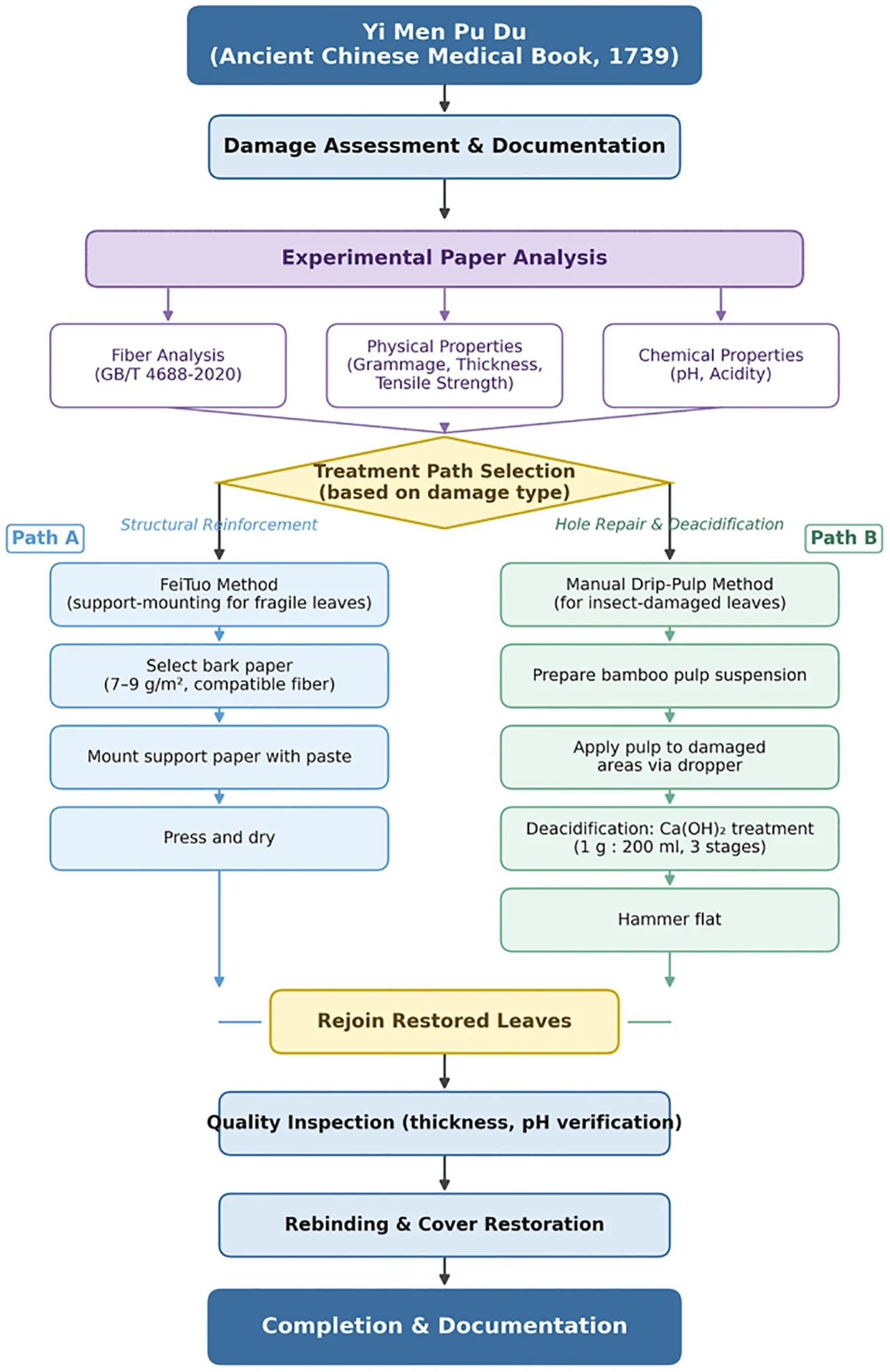
Restoration workflow of *Yi Men Pu Du.*

The FeiTuo method was employed for leaves with thin paper and weakened structural integrity—specifically when the paper could not be lifted without sustaining further damage. In this technique, a thin support paper was mounted onto the damaged leaf using paste, reinforcing the overall structure. The manual drip-pulp method involved shredding repair paper and suspending it in water to form a dilute pulp slurry, which was then carefully applied to damaged areas using a dropper. A principal advantage of this method is that it does not materially increase paper thickness, making it particularly suitable for ancient books with densely distributed insect holes.

Repair papers were selected based on fiber compatibility with the original substrate (as confirmed by subsequent fiber analysis; Section 3.1). Fujian Sanming Xishan handmade bamboo raw paper was selected for drip-pulp repair, while Zhejiang Ningbo handmade bark paper (a swallow-bark and mulberry-bark blend, 7–9 g, in two batches) was chosen as the support paper for the FeiTuo method. Leather-bound cover paper of matching color from the library’s collection was used for cover restoration.

### 2.3. Sample overview

All experimental samples were derived from the original leaves and repair papers of *Yi Men Pu Du*. The 22 sample numbers correspond to 22 distinct experiments; some physical paper specimens were tested in multiple experiments and thus carry different sample numbers. For example, Samples 1 and 9 represent the same physical paper tested for grammage and tensile strength, respectively; Samples 3, 11, and 19 represent the same bamboo paper tested for grammage, tensile strength, and pH. The complete sample mapping is presented in Table 2.

**Table 2:** Overview of experimental samples.

Experiment type	Sample no.	Paper type and purpose	Key data
Physical — Grammage (Table 3)	Sample 1	Support paper 1, bark paper	19.768 g/m²
	Sample 2	Support paper 2, bark paper	19.336 g/m²
	Sample 3	Drip-pulp paper, bamboo paper	18.623 g/m²
Physical — Thickness (Table 4)	Sample 4	Pre-restoration book leaf 1	88 μm
	Sample 5	Pre-restoration book leaf 2	58 μm
	Sample 6	Post-restoration leaf (FeiTuo method)	99 μm
	Sample 7	Post-restoration leaf (drip-pulp method)	195 μm
	Sample 8	Post-restoration, hammered (drip-pulp method)	185 μm
Physical — Tensile strength (Table 5)	Sample 9	Support paper 1, bark paper	877/519 N/m
	Sample 10	Support paper 2, bark paper	793/369 N/m
	Sample 11	Drip-pulp paper, bamboo paper	1252/930 N/m
Chemical — Fiber analysis (Fig 2)	Sample 12	Original book cover	100% bark fiber
	Sample 13	Guard leaf	Mixed bark fiber
	Sample 14	Text leaf	100% bamboo fiber
Chemical — Acidity (Table 6)	Sample 15	Pre-restoration book leaf 1	pH 5.1
	Sample 16	Pre-restoration book leaf 2	pH 5.4
	Sample 17	Post-restoration leaf (FeiTuo method)	pH 6.6
	Sample 18	Post-restoration leaf (drip-pulp method)	pH 5.8
	Sample 19	Drip-pulp repair paper, bamboo paper	pH 6.3
	Sample 20	Drip-pulp method, 1st alkali treatment	pH 7.4
	Sample 21	Drip-pulp method, 2nd alkali treatment	pH 7.6
	Sample 22	Drip-pulp method, 3rd alkali treatment	pH 7.7

**Fig 2:**
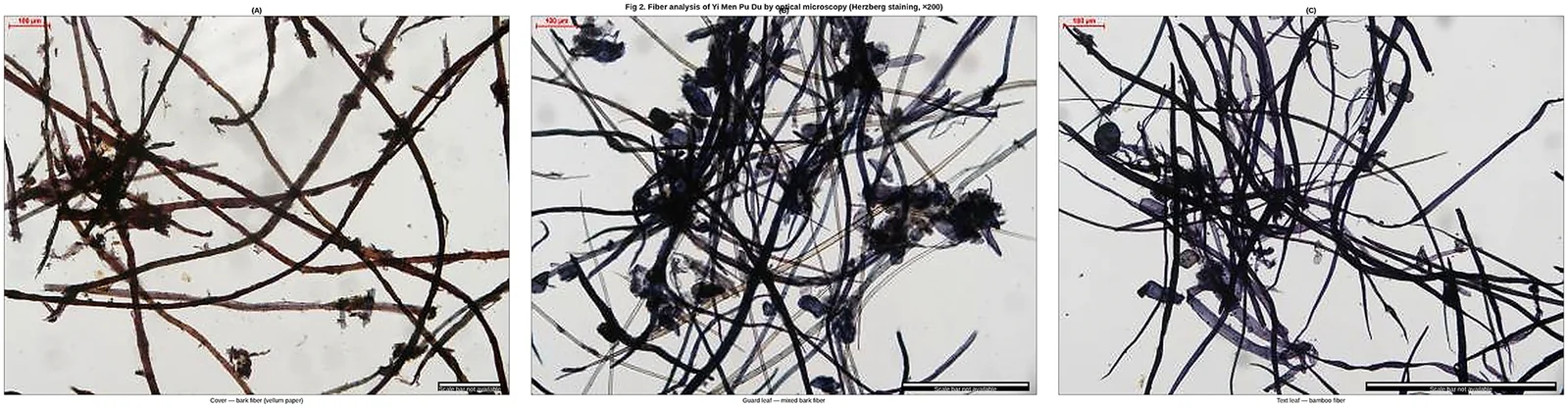
Fiber micrographs of the cover (left), guard leaf (center), and text leaf (right) of *Yi Men Pu Du.*

### 2.4. Physical property testing

Atmospheric conditions during all physical testing were maintained at 23.0 ± 1.0 °C and 50.0 ± 2.0% relative humidity. Physical property testing encompassed grammage determination, thickness measurement, and tensile strength testing.

#### 2.4.1. Grammage determination.

Grammage determination was performed on the three repair papers. Samples were collected by cutting 15 mm × 15 mm specimens from two types of support papers and the drip-pulp paper (Samples 1–3). Cutting error did not exceed 0.1 mm for each sample. The testing equipment was a Sartorius MCE6.2S-2CCN-M microbalance (readability: ≤ 0.001 mg). The testing standard was *Paper and Board — Determination of Grammage* (UNI EN ISO 536:2012) [14]. Grammage was calculated using the formula *w = g/F*, where *g* is the sample mass and *F* is the sample area.

#### 2.4.2 Thickness measurement.

Paper thickness was measured for ancient book leaves both before and after restoration. Pre-restoration samples (Samples 4 and 5) were taken from two book leaves; post-restoration samples were collected from leaves repaired using the FeiTuo method (Sample 6) and the manual drip-pulp method (Sample 7), with an additional measurement taken after hammering the drip-pulp-repaired leaf (Sample 8). The testing equipment was a TMI paper thickness gauge. The testing standard was *Paper and Board — Determination of Thickness* (GB/T 451.3-2008) [15]. Ten points were measured on each sample, and the mean thickness was calculated (n = 10 per sample).

#### 2.4.3 Tensile strength testing.

Tensile strength was measured for the three repair papers. Three 15 mm × 15 mm specimens were cut from each paper type, with a cutting error not exceeding 0.1 mm (Samples 9–11). The testing equipment was a Sichuan Changjiang DCP-KZ(W)30 paper tensile strength tester. The standard used was *Paper and Board — Determination of Tensile Properties — Constant Rate of Elongation Method (100 mm/min)* (GB/T 22898−2008) [16].

### 2.5. Chemical property testing

Chemical property testing encompassed fiber raw material identification and acidity measurement. Sample materials and experimental conditions were consistent with those described in Section 2.4.

#### 2.5.1. Fiber raw material identification.

Fiber raw material identification was performed on the cover, guard leaf, and text leaf of the original book. Three samples (Samples 12, 13, and 14), each measuring approximately 0.5 mm × 0.5 mm, were collected. The testing equipment was a Nikon 90i biological upright microscope. The standard was *Paper, Board and Pulp — Analysis of Fiber Furnish* (GB/T 4688−2020) [17]. Herzberg staining was applied to differentiate fiber types based on color response.

#### 2.5.2. Acidity determination.

Acidity testing was performed on *Yi Men Pu Du* leaves before and after restoration, as well as on the drip-pulp repair paper and deacidification treatment samples (Samples 15–22). Surface pH was measured using a Hanna HI9125 pH meter, and cold-extraction pH was determined using a Sartorius pp-15 pH meter. The testing standards were *Paper and Board — Determination of Surface pH* (GB/T 13528–2015) [18] and *Paper, Board and Pulp — Determination of Acidity or Alkalinity* (GB/T 1545–2008) [19].

### 2.6. Deacidification treatment

Calcium hydroxide (Ca(OH)_2_) was selected as the deacidifying agent. A uniform alkali solution was prepared at a concentration of 1 g Ca(OH)_2_ per 200 ml purified water and applied in three successive stages:

- **Stage i:** The alkaline solution was introduced into a water tray at the above concentration. Book leaves were placed on a bamboo curtain aligned with the grain direction and gently submerged until fully soaked. The bamboo curtain and leaves were then removed together. This constituted the first alkali contact (Sample 20).
- **Stage ii:** During the drip-pulp process, pre-mixed alkaline solution was incorporated into the pulp slurry at a total amount of 5 g Ca(OH)_2_ in 400 ml—representing the application of the same 1 g:200 ml concentration to a larger working volume. This constituted the second alkali contact for each leaf (Sample 21).
- **Stage iii:** During the repair process, a spray bottle was used to continuously supplement the same alkali solution, ensuring close adhesion between the book leaves and the repair curtain. This constituted the third alkali treatment (Sample 22).

For the FeiTuo method, the alkaline solution at 1 g:200 ml was added directly to the diluted paste water used for mounting, providing deacidification in a single step.

## 3. Results

### 3.1. Fiber analysis

As shown in Fig 2, the book cover fibers were relatively long with thick, knob-like thickened fiber walls and a transparent gelatinous coating on the outer wall. Herzberg staining produced a Burgundy color. The composition was identified as 100% bark fiber, consistent with vellum paper (Sample 12). The guard leaf (Sample 13) showed mixed bark fiber. For the text pages (Sample 14), the fibers were thin and short with pointed ends accompanied by nodal thickening. Microscopic examination revealed wide vessel elements with slightly inclined end walls and regularly arranged pits on the vessel wall. The parenchyma cells were rectangular with relatively uniform dimensions. The composition was identified as 100% bamboo fiber, confirming the text leaves as bamboo paper.

The fiber analysis results directly informed the selection of repair papers: Fujian Sanming Sanjue Xishan handmade bamboo raw paper was selected for drip-pulp repair to match the bamboo fiber composition of the text leaves, Zhejiang Ningbo handmade bark paper (swallow-bark and mulberry-bark blend, 7–9 g) was chosen for the FeiTuo method to match the bark fiber cover, and matching leather-bound cover paper from the library’s collection was used for cover restoration.

### 3.2. Grammage and thickness

Grammage determination of the three repair papers is presented in Table 3. All papers fell within a narrow range of 18.623–19.768 g/m^2^.

**Table 3:** Grammage determination of repair papers.

Sample	Description	Grammage (g/m²)
Sample 1	Support paper 1, bark paper	19.768
Sample 2	Support paper 2, bark paper	19.336
Sample 3	Drip-pulp paper, bamboo paper	18.623

Thickness measurement results are presented in Table 4. The difference between Samples 4 (88 μm) and 5 (58 μm) before repair indicated variability in the original paper thickness. Based on the process characteristics of the two methods, leaves with thicker and more stable structure were selected for the drip-pulp method, while thinner leaves with overall frame damage and numerous fragments were assigned to the FeiTuo method. Notably, Sample 7 (195 μm) did not reflect the expected advantage of the drip-pulp method in minimizing thickness increase. After hammering, Sample 8 showed only a marginal reduction to 185 μm. The restoration team traced the excessive thickness to two causes: (i) the initial pulping ratio of 5 g paper per 400 ml purified water produced an overly concentrated pulp suspension; and (ii) excessively wide and thick edges were created between the drip-pulp application and the original leaf margins. Technical improvements were implemented by reducing the pulping ratio to 0.5 g paper per 400 ml water and narrowing the overlap at damaged areas and edge positions. Following these adjustments, post-restoration thickness was substantially reduced and fell within an acceptable range.

**Table 4:** Thickness measurement results (mean, n = 10 per sample).

Sample	Description	Mean thickness (μm)
Sample 4	Pre-restoration book leaf 1	88
Sample 5	Pre-restoration book leaf 2	58
Sample 6	Post-restoration leaf (FeiTuo method)	99
Sample 7	Post-restoration leaf (drip-pulp method)	195
Sample 8	Post-restoration, hammered (drip-pulp method)	185

### 3.3. Tensile strength

The tensile strength results are presented in Table 5. The bamboo paper (Sample 11) exhibited 29.95%–36.66% higher longitudinal tensile strength and 44.19%–60.32% higher transverse tensile strength compared with the bark papers (Samples 9 and 10). This superior mechanical performance, combined with the fiber compatibility confirmed by microscopy, validated the selection of bamboo paper for drip-pulp repair.

**Table 5:** Tensile strength of repair papers.

Sample	Description	Tensile strength, longitudinal/transverse (N/m)
Sample 9	Support paper 1, bark paper	877/519
Sample 10	Support paper 2, bark paper	793/369
Sample 11	Drip-pulp paper, bamboo paper	1252/930

### 3.4. Acidity and deacidification

The acidity test results are presented in Table 6. Pre-restoration samples (Samples 15 and 16) showed surface pH values of 5.1 and 5.4, respectively—significantly below the acceptable limit for long-term preservation. The American Society for Testing and Materials (ASTM) specifies a minimum pH of 5.5 for the LE-50 grade, corresponding to a medium lifespan of approximately 50 years. Post-restoration, Sample 17 (FeiTuo method) showed a surface pH of 6.6, while Sample 18 (drip-pulp method) showed only 5.8. The lower post-restoration pH for the drip-pulp method is attributable to the inherent acidity of the bamboo pulp suspension. Sample 19 (drip-pulp paper alone) yielded a cold-extraction pH of 6.3. According to *Information and Documentation — Paper for Documents — Requirements for Permanence* (GB/T 24423−2009) [20], permanent paper should have a minimum alkaline reserve of 0.4 mol/kg and a cold-extraction pH within the range of 7.5–10.0. These results confirmed that targeted deacidification was necessary.

**Table 6:** Acidity test results of paper samples.

Sample	Description	pH value
Sample 15	Pre-restoration book leaf 1	5.1
Sample 16	Pre-restoration book leaf 2	5.4
Sample 17	Post-restoration leaf (FeiTuo method)	6.6
Sample 18	Post-restoration leaf (drip-pulp method)	5.8
Sample 19	Drip-pulp repair paper, bamboo paper	6.3
Sample 20	Drip-pulp method, 1st alkali treatment	7.4
Sample 21	Drip-pulp method, 2nd alkali treatment	7.6
Sample 22	Drip-pulp method, 3rd alkali treatment	7.7

The progressive deacidification protocol demonstrated a clear dose-response relationship: each successive alkali treatment yielded incrementally higher pH values (7.4 → 7.6 → 7.7). The progressive increase in pH from 5.1–5.4 (pre-restoration) to 7.4–7.7 (post-deacidification) confirmed the effectiveness of the Ca(OH)_2_ treatment.

## 4. Discussion

### Fiber analysis as a guide for repair paper selection

The fiber composition analysis of *Yi Men Pu Du* proved instrumental in guiding material selection for restoration. The identification of 100% bamboo fiber in the text leaves provided a direct scientific basis for selecting Fujian Sanming Xishan bamboo paper as the drip-pulp material, while the 100% bark fiber composition of the cover informed the choice of Zhejiang Ningbo bark paper for the FeiTuo method. This material matching approach—ensuring fiber compatibility between original and repair materials—is fundamental to achieving the restoration principle of “preserving the old as it was.” Beyond material matching, fiber analysis can also provide corroborating evidence for dating ancient paper, as fiber composition and manufacturing techniques vary across historical periods [17]. Previous studies have similarly demonstrated that fiber identification is a critical first step in paper conservation, enabling conservators to avoid incompatible repair materials that may accelerate degradation [10,21].

### pH data and deacidification effectiveness

The acidity measurements revealed severe acidification in the original book leaves (pH 5.1–5.4), well below the threshold for long-term preservation. These values are consistent with published data on acidified ancient Chinese books from the Qing dynasty period [4]. The progressive deacidification protocol demonstrated effective neutralization without over-alkalinization. The final pH range of 7.4–7.7 falls within or near the 7.5–10.0 range recommended by GB/T 24423−2009 for permanent paper [20]. Notably, the post-restoration pH of the drip-pulp method leaves (5.8) was lower than that of the FeiTuo method leaves (6.6), attributable to the inherent acidity of the bamboo pulp suspension. This finding underscores the necessity of targeted deacidification when employing the drip-pulp method, as the repair material itself can introduce additional acidity to the already-compromised original paper. The use of Ca(OH)_2_ as the deacidifying agent is well-established in paper conservation [8,9], and the concentration employed here (1 g:200 ml) proved effective in raising pH to the target range without inducing visible adverse effects such as alkaline darkening or fiber swelling.

### Pulp concentration optimization

The thickness measurements revealed a critical issue with the initial pulp concentration: the original ratio of 5 g paper per 400 ml water produced post-restoration leaves measuring 195 μm, far exceeding the pre-restoration thickness of 58–88 μm. Even after mechanical hammering, the thickness was reduced only marginally to 185 μm. The tenfold reduction in pulp concentration from 5 g/400 ml to 0.5 g/400 ml directly addressed this problem, yielding post-restoration leaves of acceptable thickness. This optimization process illustrates how quantitative monitoring during restoration—rather than reliance on empirical judgment alone—can identify and correct process deficiencies in real time. The magnitude of the concentration adjustment (10-fold reduction) reflects the non-linear relationship between pulp concentration and deposited paper thickness, a factor that warrants further systematic investigation. Although intermediate gradient testing was not conducted, the practical outcome was satisfactory, and future studies could establish quantitative concentration–thickness curves for different paper types.

### Physical property testing supporting method selection

The tensile strength results provided quantitative evidence for the selection of bamboo paper as the drip-pulp material. The bamboo paper (Sample 11) exhibited 29.95%–36.66% higher longitudinal tensile strength and 44.19%–60.32% higher transverse tensile strength compared with the bark papers (Samples 9 and 10). Research has demonstrated that bamboo pulp paper exhibits a higher breaking index than wood pulp paper, attributable to the higher content of long-fiber and fine-fiber components in bamboo pulp, which reduces pulp freeness and improves water retention capacity [22]. Additionally, under comparable beating conditions, bamboo pulp fibers are more readily cut and prone to kinking than wood pulp fibers, resulting in a higher tensile index, tearing index, and breaking index [22]. After secondary beating, the bamboo paper used in this study showed substantially enhanced fiber properties compared with raw bamboo pulp. The grammage data further supported the material selection, as all three repair papers fell within a narrow range (18.623–19.768 g/m²), ensuring minimal weight discrepancy between original and repaired areas.

### Integration of experimental techniques into traditional restoration workflows

This case study demonstrates the feasibility and value of embedding systematic experimental analysis into traditional ancient book restoration practice. The integration operated at multiple levels: (i) pre-restoration fiber analysis guided material selection, replacing subjective judgment with objective data; (ii) pH testing identified the need for deacidification and enabled monitoring of treatment effectiveness; (iii) thickness measurements provided quantitative feedback on repair quality, enabling real-time process optimization; and (iv) tensile strength testing validated the mechanical suitability of selected repair materials. The successful outcome suggests that a standardized testing protocol—incorporating fiber microscopy, pH measurement, grammage determination, thickness monitoring, and tensile strength testing—could be adopted by restoration laboratories as a routine quality assurance framework, particularly for high-priority conservation projects.

### Limitations

Several limitations of this study should be acknowledged. First, the sample size was constrained by the single-case nature of the project, as destructive sampling of a valuable ancient book is necessarily limited. Thickness measurements were taken at ten points per sample, but individual measurement data were not available for calculating standard deviations; future studies should report full descriptive statistics. Second, as a single-case study of one ancient book, the generalizability of the specific findings is limited; however, the methodological framework is transferable. Third, no formal statistical tests (e.g., t-tests or ANOVA) were applied to compare pre- and post-treatment measurements due to the limited sample sizes. Fourth, the long-term effects of the Ca(OH)_2_ deacidification treatment on paper properties—including potential alkali migration and its impact on fiber structure—were not evaluated and would require accelerated aging studies [23]. Fifth, the pulp concentration optimization was conducted as a single step change (5 g to 0.5 g per 400 ml) without intermediate gradient testing, which precludes determination of the optimal concentration range.

## 5. Conclusions

This study demonstrates that systematic experimental paper analysis can effectively guide the restoration of ancient books, transforming what has traditionally been an experience-based craft into an evidence-based practice. The principal findings are as follows:

1. **Fiber analysis guided material selection.** Microscopic fiber identification revealed that the cover of *Yi Men Pu Du* was composed of 100% bark fiber (vellum paper) while the text leaves were 100% bamboo fiber, providing a direct scientific basis for selecting matched repair papers.
2. **Deacidification effectively neutralized paper acidity.** Pre-restoration surface pH values of 5.1–5.4 indicated severe acidification. A progressive Ca(OH)_2_ deacidification protocol (1 g:200 ml concentration) raised the pH to 7.4–7.7, bringing the paper within or near the range recommended for permanent preservation.
3. **Pulp concentration optimization resolved thickness issues.** Quantitative thickness monitoring identified excessive post-restoration thickness attributable to overly concentrated pulp. Reducing the pulp ratio from 5 g/400 ml to 0.5 g/400 ml effectively resolved this issue while maintaining repair quality.
4. **Tensile strength testing validated repair material performance.** The bamboo repair paper exhibited 29.95%–36.66% higher longitudinal and 44.19%–60.32% higher transverse tensile strength than the bark papers, confirming its suitability for the drip-pulp method.
5. **The restored edition holds significant bibliographic value.** The edition of *Yi Men Pu Du* restored in this project predates the previously known earliest edition (1786, engraved by Li Shui, Chen Yuan, and Qian Yi Tang at Bao Tian Zhai during the 51st year of the Qianlong reign) by 47 years [24], representing the earliest known edition of this important medical text.

These findings support the broader adoption of experimental testing protocols in ancient book restoration laboratories, enabling conservators to make informed, data-driven decisions regarding repair materials and methods.
