# Supplementary material for: Integrating experimental paper analysis into traditional ancient book restoration: A case study of the Chinese medical text Yi Men Pu Du
Source: PLoS One. 2026 Oct 9;21(10):e0360242. doi: 10.1371/journal.pone.0360242 (PMC13652232; doi:10.1371/journal.pone.0360242)
Supplement: Supplementary file 1 [file pone.0360242.s001.docx]

Dear Editor,

The article has been revised according to the requirements of the experts, and the specific modifications are as follows:

1.Manuscript meets PLOS ONE's style requirements.

2.The ‘Funding Information’ and ‘Financial Disclosure’ sections do match.

3.Write "All data are in the manuscript and/or supporting information files" as my Data Availability Statement.

4. Including fig1 and fig2 in the attachment.

5. Not related to this content.

6.The font now is Times New Roman.

7.’Response to Reviewers’, ’Revised Manuscript with Track Changes’, ’Manuscript’ are submitted.

Sincerely,

Li Meng
